# Supplementary material for: Efficiency of RAPD and SCoT Markers in the Genetic Diversity Assessment of the Common Bean
Source: Plants (Basel). 2023 Jul 25;12(15):2763. doi: 10.3390/plants12152763 (PMC10420954; doi:10.3390/plants12152763)
Supplement: Supplementary file 1 [file plants-12-02763-s001.zip › plants-2497609-supplementary.pdf]

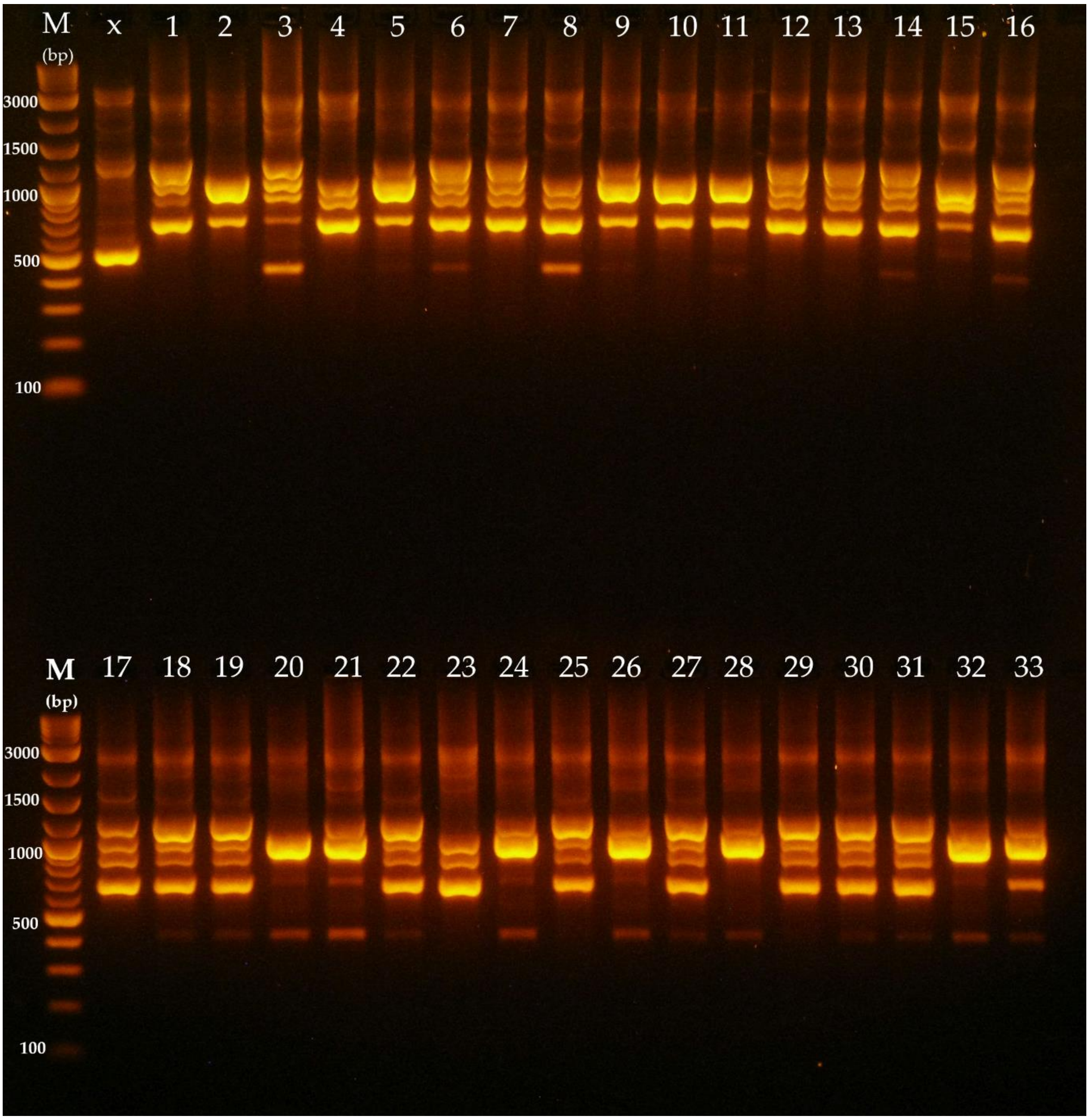

Figure S1. RAPD-PCR amplification products of the common bean genotypes generated by marker OPC-13. Notes: M - DNA ladder; x – sample is not included in the collection pf genotypes analysed in the work; 1-33 - common bean genotypes listed in Table 4 under the numbers 1-33.

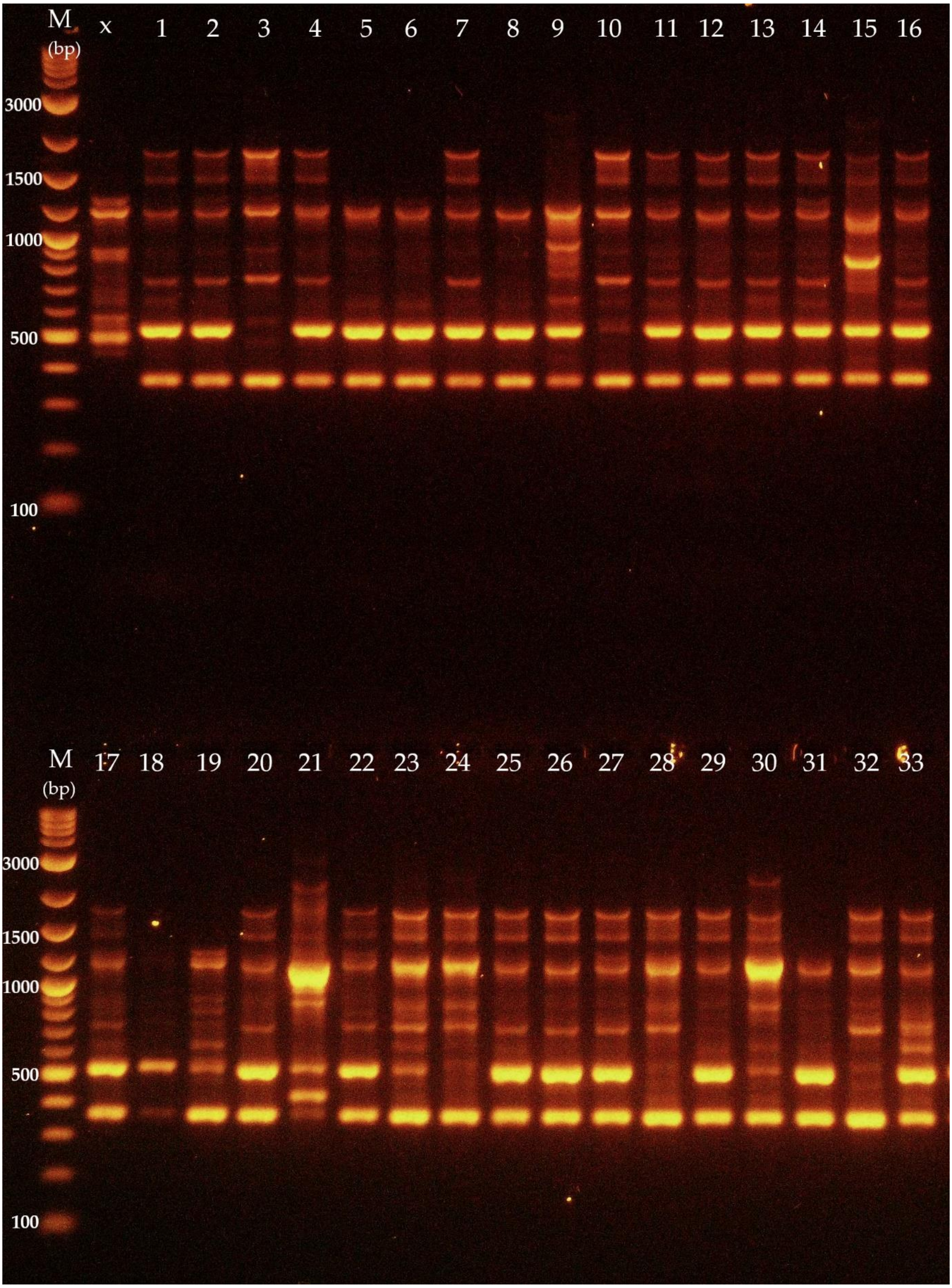

Figure S2. SCoT-PCR amplification products of common bean genotypes generated by marker SCoT54. Notes: M - DNA ladder; x – sample is not included in the collection pf genotypes analysed in the work; 1-33 - common bean genotypes listed in Table 4 under the numbers 1-33.
